# Supplementary material for: Rapid mechanochemical synthesis of polyanionic cathode with improved electrochemical performance for Na-ion batteries
Source: Nat Commun. 2021 May 14;12:2848. doi: 10.1038/s41467-021-23132-w (PMC8121810; doi:10.1038/s41467-021-23132-w)
Supplement: Supplementary file 1 — Supplementary Information [file 41467_2021_23132_MOESM1_ESM.pdf]

Supplementary information for

Rapid mechanochemical synthesis of polyanionic cathode with superior performance for Na-ion batteries

Xing Shen[1,4], Quan Zhou[2], Miao Han[2], Xingguo Qi[2], Bo Li[1], Qiangqiang Zhang[2], Junmei Zhao[1,3,4*], Chao Yang[1], Huizhou Liu[1] and Yong-Sheng Hu[2*]

[1]CAS Key Laboratory of Green Process and Engineering, State Key Laboratory of Biochemical Engineering, Institute of Process Engineering, Chinese Academy of Sciences, Beijing 100190, China *E-mail: jmzhao@ipe.ac.cn

[2]Key Laboratory for Renewable Energy, Beijing Key Laboratory for New Energy Materials a nd Devices, Beijing National Laboratory for Condensed Matter Physics, Institute of Physics, Chinese Academy of Sciences, Beijing 100190, China *E-mail: yshu@iphy.ac.cn

[3]Innovation Academy for Green Manufacture, Chinese Academy of Sciences, Beijing 100190, China

[4] School of Chemical Engineering, University of Chinese Academy of Sciences, Beijing 100049, China

Supplementary Note 1

The calculation of pseudocapacitance contribution and D_{Na^+}

The pseudocapacitance contribution can be calculated based on the following methods, here we take calculation process of the perudocapacitive contributions at 0.9 mV s^{-1} for example. First, get CV curves at various scan rate (**Supplementary Fig. 17a**). Upon the same voltage range, the current at various scan rates was obtained and k_1 value (the slope value of the curve) can be calculated according to the **Supplementary Equation (1)**:

$$i/v^{0.5} = k_1 v^{0.5} + k_2 \qquad \textbf{Supplementary Equation (1)}$$

and then, get the k_1 value at different potentials. Finally, we can get the capacitive contribution part current values at different potentials by $k_1 v$ (**Supplementary Fig. 19**).

Supplementary Note 2

Supplementary Equation (2) is used to determine the diffusion coefficient of Na ions derived from Weppner et al, and the parameters needed can be obtained from GITT curves.

$$D_{Na^+} = \frac{4}{\pi\tau}\left(\frac{m_B V_M}{M_B S}\right)^2 \left(\frac{\Delta E_s}{\Delta E_\tau}\right)^2 \qquad (\tau \ll L^2/D_{Na^+})$$ **Supplementary Equation (2)**

Where m_B and M_B are the mass and molecular weight of $Na_3(VOPO_4)_2F$, respectively. V_M is the molar volume of $Na_3(VOPO_4)_2F$. τ is the time for an applied galvanostatic current density. S is the active surface of the electrode. L is the average radius of the material particles. ΔE_s and ΔE_τ are the quasi-equilibrium potential and the change of cell voltage during the current pulse, respectively.

Supplementary Table 1. Mole ratios of four types starting materials for ten different reactions

No.	Vanadium source	Optimal phosphorus source	Fluorine source	Reductant	mole ratio	pH values of filtrate
1	V_2O_3	H_3PO_4	NaF		V:P:F=1:6:3.4	3.52
2	VCl_3	$NaH_2PO_4 \cdot 2H_2O$	NaF		V:P:F=1:3:1.7	2.85
3	$V(acac)_3$	$NaH_2PO_4 \cdot 2H_2O$	NaF		V:P:F=1:3:1.7	4.39
4	VO_2	H_3PO_4	NaF		V:P:F=1:3:1.7	3.10
5	$VOSO_4$	$NaH_2PO_4 \cdot 2H_2O$	NaF		V:P:F=1:3:1.7	5.32
6	$VO(acac)_2$	$NaH_2PO_4 \cdot 2H_2O$	NaF		V:P:F=1:3:1.7	5.52
7	V_2O_5	H_3PO_4	NaF	$HONH_2 \cdot HCl$	V:P:F:R=1:3:1.8:6	3.65
8	NH_4VO_3	$NaH_2PO_4 \cdot 2H_2O$	NaF	$HONH_2 \cdot HCl$	V:P:F:R=1:1.5:1:3	5.72
9	$NaVO_3$	$NaH_2PO_4 \cdot 2H_2O$	NaF	$HONH_2 \cdot HCl$	V:P:F:R=1:1.5:1:3	5.43
10	Na_3VO_4	$NaH_2PO_4 \cdot 2H_2O$	NaF	$HONH_2 \cdot HCl$	V:P:F:R=1:1.5:1:3	5.86

Supplementary Table 2. The current market price of starting materials. (The price data was collected from the commonly used purchasing website https://china.guidechem.com or https://www.1688.com)

Vanadium source	Unit price (RMB kg^{-1})	Product purity (%)	Suppliers
V_2O_3	480	99.0	Hubei Xinrunde chemical Co. LtD
VCl_3	750	99.0	Hubei Xinrunde chemical Co. LtD
$V(acac)_3$	650	99.0	Yunnan Lilian biology Co. Ltd
VO_2	230	99.0	Hubei Xinrunde chemical Co. LtD
$VOSO_4$	260	99.0	Wuxi Zhanwang chemical Co. LtD
$VO(acac)_2$	600	99.0	Liyang Kaixin new material technology Co. LtD
V_2O_5	180	99.5	Wuxi Zhanwang chemical Co. LtD
NH_4VO_3	140	99.5	Wuxi Zhanwang chemical Co. LtD
$NaVO_3$	110	99.5	Shandong xiangguan new material Co. LtD
$Na_3VO_4·16H_2O$	40	99.5	Zhengzhou Hengxiang chemical Co. LtD
$NaH_2PO_4·2H_2O$	3.8	98%	Jinan honghxiang chemical Co. LtD

H_3PO_4	5.0	85%	Guangxi xinfubei technology Co. LtD
NaF	5.8	98%	Nanjing mizhao chemical Co. LtD
$HONH_2 \cdot HCl$	18	99%	Deli chemical Co. LtD

Supplementary Table 3. The preparation costs of starting materials for NVPFs from different vanadium sources

Vanadium source	Mole ratio	Cost of 1kg NVPFs (RMB)
V_2O_3	V:P:F=1:6:3.4	187.0
VCl_3	V:P:F=1:3:1.7	580.4
$V(acac)_3$	V:P:F=1:3:1.7	1103.8
VO_2	V:P:F=1:3:1.7	100.3
$VOSO_4$	V:P:F=1:3:1.7	218.6
$VO(acac)_2$	V:P:F=1:3:1.7	791.2
V_2O_5	V:P:F:R=1:3:1.8:6	105.1
NH_4VO_3	V:P:F:R=1:1.5:1:3	103.9
$NaVO_3$	V:P:F:R=1:1.5:1:3	89.4
$Na_3VO_4\cdot 16H_2O$	V:P:F:R=1:1.5:1:3	113.7

Supplementary Table 4. The yields obtained at different mole ratios of starting materials (V, P and F represents $NaVO_3$, $NaH_2PO_4 \cdot 2H_2O$ and NaF, respectively)

V:P:F	Yield
1:1:0.5	78.43%
1:1.5:0.5	84.67%
1:1.5:1	94.07%
1:1.5:1.5	94.12%
1:2.5:0.5	88.79%
1:2.5:1	94.02%
1:2.5:1.5	94.58%

Supplementary Table 5. Crystallographic and Rietveld refinement parameters of as-synthesized $Na_3(VOPO_4)_2F$ compound

Formula	$Na_3(VOPO_4)_2F$
Crystallographic system	Tetragonal
Space group	*I4/mmm* (139)
	a=6.3884(1) Å
	b=6.3884(1) Å
Cell parameters	c=10.6582(2) Å
	α=β=γ=90°
	V=434.98(1) Å^3
	R_{wp}=7.43%
Reliability factors	R_p=8.51%
	x^2=1.87
Crystalline size	5 nm

Supplementary Table 6. Atomic coordinates, cation and anion occupancies of as-synthesized $Na_3(VOPO_4)_2F$ compound

Element type	Fractional coordinates x	y	z	Mult.	Occu.	Uiso
Na1	-0.2548(6)	-0.2548(6)	0	8	0.832(5)	0.0233(4)
Na2	-0.3186(2)	-0.2370(1)	0	16	0.143(6)	0.0022(3)
V1	0	0	0.1991(3)	4	1	0.0155(2)
P1	0.5	0	0.25	4	1	0.1674(1)
F1	0	0	0	2	1	0.0052(6)
O1	0.3082(9)	0	0.1630(9)	16	1	0.0177(9)
O2	0	0	0.3527(2)	4	1	0.0000(9)

Supplementary Table 7. The accurate weight percents of KB in the NVOPF/KB samples

Sample number	C content (wt%)
NVOPF/6%KB	5.98
NVOPF/8%KB	8.03
NVOPF/10%KB	9.89
NVOPF/12%KB	10.95

Supplementary Table 8 The comparison of synthesis and electrochemical performance for $Na_3(VOPO_4)_2F$ between the present literatures and this work

**Material**	**Synthetic method**	**Vanadium source**	**Reaction condition**	**Half-cell performance**	**Full-cell performance**
NVOPF[1]	Two-step solid-state method	V_2O_5	850 °C 8 h; 750 °C 1 h	87 mAh g^{-1} at 0.1 C	
NVOPF@C[2]	single-step hydrothermal treatment	V_2O_3	170 °C 65 h	102 mAh g^{-1} at 0.2 C, 68 mAh g^{-1} at 1 C	
NVOPF@KB[3]	Ceramic-hydrothermal synthesis	V_2O_5	850 °C 8 h for ceramic method; 170 °C 65 h for hydrothermal		
NVOPF[4]	phase-transfer assisted solvo-thermal strategy	$VO(acac)_3$	120 °C 10 h	120 mAh g^{-1} at 0.2 C (no measurable loss over 50 cycles)	
RuO_2-coated NVOPF[5]	microemulsion-mediated hydrothermal synthesis	NH_4VO_3	180 °C 24 h	128 mAh g^{-1} at 0.1 C; 105 mAh g^{-1} at 20 C (90.5%, 1000 cycles)	
NVOPF[6]	solvothermal low-temperature method	$VO(acac)_3$	120 °C 10 h	123.5 mAh g^{-1} at 0.1 C; 103 mAh g^{-1} at 2 C (90%, 1200 cycles)	

NVOPF@ Graphene[7]	Solvothermal-microemulsion-calcination method	NH_4VO_3	180 ºC 20 h for solvotherma lmethod; 400 ºC 35 min for calcination	123.5 mAh g^{-1} at 0.1 C; 70 mAh g^{-1} at 45 C (81%, 2000 cycles)	
NVOPF[8]	hydrothermal method	V_2O_5	170 ºC 12 h	127.8 mAh g^{-1} at 0.1 C; 92.3 mAh g^{-1} at 20 C (80.9%, 2000 cycles)	NVOPF-NTP‖pre-sodiated Sb-CNT 120.3 mAh g^{-1} at 1 C; 96.2 mAh g^{-1} at 5 C (80%, 50 cycles)
NVOPF@rGO[9]	solid state reaction followed by a hydrothermal method	V_2O_5	170 ºC 72 h for solvotherma l method; 850 ºC 6 h for calcination	108 mAh g^{-1} at 0.1 C (98%, 250 cycles)	NVOPF@rGO‖$NaTi_2(PO_4)_3$(NTP)-MWCNT 98 mAh g^{-1} at 0.2 C; 64 mAh g^{-1} at 2 C (84%, 1000 cycles)
Ru-doped NVOPF with RuO_2 coating[10]	low-temperature solvothermal method	NH_4VO_3	170 ºC 24 h	116.8 mAh g^{-1} at 0.5 C; 102.5 mAh g^{-1} at 20 C (90.2%, 1000 cycles)	
NVOPF/rGO[11]	spray-drying method and subsequent calcination process	NH_4VO_3	400 ºC 5 h for calcination	127.2 mAh g^{-1} at 0.2 C; 87.2 mAh g^{-1} at 30 C (83.4%, 2000 cycles)	

NVOPF[12]	two-step solvothermal synthesis	NH_4VO_3	180 ºC 6 h	130 mAh g^{-1} at 0.5 C; 63.2 mAh g^{-1} at 50 C (72%, 10000 cycles)	NVOPF‖pre-sodiated VO_2 98 mAh g^{-1} at 4 C (80%, 220 cycles)
NVOPF@C[13]	low-temperature hydrothermal reaction	V_2O_5	160 ºC 12 h	116.4mAh g^{-1} at 0.2 C; 87.4 mAh g^{-1} at 10 C (82.1%, 1200 cycles)	
NVOPF[14]	Hydrothermal method	$VOSO_4$	120 ºC 10 h	128 mAh g^{-1} at 0.15 C; 120 mAh g^{-1} at 1.5 C (87.5%, 120 cycles)	NVOPF/$Na_2C_4O_4$‖hard carbon 120 mAh g_{-1} at 0.05 C; 90 mAh g^{-1} at 2 C
NVOPF@C[15]	mild hydrothermal	VO_2	170 ºC 65 h	123.5 mAh g^{-1} at 0.05 C; 81.2 mAh g^{-1} at 10 C	
NVOPF[16]	Room-temperature synthesis	$NaVO_3$	RT, 144 h	111 mAh g^{-1} at 0.1 C; 81 mAh g^{-1} at 15 C (70%, 3000 cycles)	
NVOPF[17]	Hydrothermal method	$VOSO_4$	120 ºC 10 h	115.3 mAh g^{-1} at 0.2 C; 101.6 mAh g^{-1} at 10 C (86%, 1500 cycles)	
NVOPF[18]	hydrothermal method	V_2O_5	170 ºC 12 h	123.2 mAh g^{-1} at 0.1 C; 116 mAh g^{-1} at 1 C (94.8%, 500 cycles)	
NVOPF@C[19]	microwave-assisted refluxing synthesis	$VO(acac)_3$	120 ºC 1 h	127.9 mAh g^{-1} at 0.1 C; 95.3 mAh g^{-1} at 20 C (82.1%, 2000 cycles)	
Fe^{3+}-substituted NVOPF[20]	sol−gel approach	$VO(acac)_3$	300 ºC 3 h	86 mAh g^{-1} at 0.1 C; 58 mAh g^{-1} at 0.5 C	
TiO_2-coating NVOPF[21]	sol-gel method combined with high temperature	NH_4VO_3	45 ºC 15 h for sol-gel process, 500	118 mAh g^{-1} at 0.1 C; 68 mAh g^{-1} at 1 C (73.5%, 500 cycles)	

	calcination		ºC 6 h for calcination		
NVOPF@C[22]	Hydrothermal-HEBM	$VOSO_4$	180 ºC 10 h	128.7 mAh g^{-1} at 0.2 C; 62.1 mAh g^{-1} at 150 C (71%, 12000 cycles)	NVOPF@C/$Na_2C_4O_4$‖NTP 115.4 mAh g^{-1} at 0.2 C; 81.9 mAh g^{-1} at 50 C (71%, 5000 cycles)
NVOPF@rGO[23]	solvothermal and electrostatic spray deposition	$VO(acac)_3$	180 ºC 16 h for solvothermal process	115 mAh g^{-1} at 1 C; 106 mAh g^{-1} at 20 C (76%, 1000 cycles)	NVOPF@rGO‖$Na_3V_2(PO_4)_3$(NVP)@rGO 87 mAh g^{-1} at 1 C; 84 mAh g^{-1} at 10 C (86%, 100 cycles)
NVOPF@CNF[24]	Hydrothermal-calcination process	NH_4VO_3	180 ºC 20 h for hydrothermal process, 400 ºC 1 h for calcination	120 mAh g^{-1} at 0.2 C; 78 mAh g^{-1} at 5 C (85%, 500 cycles)	
NVOPF@C[25]	one-step hydrothermal strategy	$NaVO_3$	180 ºC 24 h	120 mAh g^{-1} at 0.2 C; 102 mAh g^{-1} at 2 C (90.36%, 500 cycles)	
Fe-doped NVOPF[26]	Hydrothermal method	$VO(acac)_3$	175 ºC 72 h	102.3 mAh g^{-1} at 0.05 C; 85 mAh g^{-1} at 0.5 C	
NVOPF/graphene aerogels[27]	Hydrothermal-modified Hummers method	V_2O_5	170 ºC 12 h for hydrothermal process, 70 ºC 8 h for	135.4 mAh g^{-1} at 0.2 C; 62.1 mAh g^{-1} at 40 C (100%, 1000 cycles)	NVOPF@GAs‖Sb/G/C 84.5 mAh g^{-1} at 2 C (97%, 100 cycles)

			freeze-drying		
NVPF/C[28]	sol–gel method	NH_4VO_3	120 ºC 12 h for sol-gel process, 650 ºC 8 h for calcination	130 mAh g^{-1} at 0.5 C; 57 mAh g^{-1} at 30 C (50%, 3000 cycles)	
NVPF/rGO[29]	sol–gel method	NH_4VO_3	600 ºC 6 h for calcination	109 mAh g^{-1} at 0.5 C; 99 mAh g^{-1} at 10 C (75%, 3000 cycles)	
NVPF/C@rGO[30]	sol–gel method	NH_4VO_3	80 ºC 12 h for sol-gel process, 650 ºC 8 h for calcination	124.5 mAh g^{-1} at 0.1 C; 119.2 mAh g^{-1} at 1 C (90.9%, 700 cycles)	
NVPF/KB[31]	Solvothermal–Ball-Milling Method	V_2O_5	180 ºC 12 h for sol-gel process, 580 rpm 1 h for ball milling	138 mAh g^{-1} at 0.5 C; 134 mAh g^{-1} at 1 C (86.4%, 300 cycles)	
NVPF/CNT[32]	spray-drying	V_2O_5	600 ºC 2 h for calcination	125 mAh g^{-1} at 0.1 C; 134 mAh g^{-1} at 1 C (97.5%, 100 cycles)	
Y-doped NVPF[33]	sol–gel method	NH_4VO_3	75 ºC 12 h for sol-gel process, 750	124.1 mAh g^{-1} at 0.1 C; 118.2 mAh g^{-1} at 1 C (93.46%, 200 cycles)	

			°C 8 h for calcination		
C/Al_2O_3-NVPF[34]	sol–gel method	V_2O_5	60 °C for sol-gel process, 700 °C 10 h for calcination	128 mAh g^{-1} at 0.1 C; 122.8 mAh g^{-1} at 1 C (95.6%, 100 cycles)	
VSC/VSU Ti-doped NVPF[35]	sol–gel method	NH_4VO_3	80 °C 12 h for sol-gel process, 750 °C 8 h for calcination	125 mAh g^{-1} at 0.2 C; 104 mAh g^{-1} at 40 C	NVPF-Ti||NVP<br>99.8 mAh g^{-1} at 0.2 C; 60 mAh g^{-1} at 40 C (91.3%, 500 cycles)
N-doped NVPF@C[36]	sol–gel method	V_2O_5	60 °C for sol-gel process, 700 °C 10 h for calcination	113.8 mAh g^{-1} at 0.2 C; 98.0 mAh g^{-1} at 10 C (95.8%, 800 cycles)	
N-doped NVPF@C[37]	high-temperature solid-state method	NH_4VO_3	600 °C 6 h	120.3 mAh g^{-1} at 0.5 C; 67.8 mAh g^{-1} at 50 C (84.8%, 2500 cycles)	NVPF-NC||HC<br>95.8 mAh g^{-1} at 0.2 C; 82.4 mAh g^{-1} at 0.5 C (87.7%, 300 cycles)
K-doped NVPF@CNT[38]	sol–gel method	NH_4VO_3	80 °C for sol-gel process, 750 °C 6 h for calcination	120 mAh g^{-1} at 1 C; 52 mAh g^{-1} at 50 C (90%, 6000 cycles)	
NVPF/C[39]	sol–gel method	NH_4VO_3	60 °C 3 h for	103 mAh g^{-1} at 0.2 C; 96 mAh g^{-1} at 5	

			sol-gel process, 750 °C 6 h for calcination	C (91.9%, 500 cycles)	
NVPF[40]	Hydrothermal method	NH_4VO_3	200 °C 12 h	124.8 mAh g^{-1} at 10 mA g^{-1}; 102.8 mAh g^{-1} at 50 mA g^{-1} (87%, 550 cycles)	
nanosized NVPF[41]	sol–gel method	NH_4VO_3	80 °C for sol-gel process, 700 °C 4 h for calcination	124 mAh g^{-1} at 0.1 C; 93% for 300 cycles at 1.0 A g^{-1}	n-NVPF‖SnPx/carbon 98 mAh g-1 at 0.1 A g^{-1}; 78% for 200 cycles at 0.1 A g−1
NVPF@graphene nanosheets[42]	Hydrothermal method	$VO(acac)_2$	200 °C 24 h	103 mAh g^{-1} at 0.2 C; 96 mAh g^{-1} at 5 C (91.9%, 500 cycles)	
NVPF@rGO[43]	Hydrothermal method	V_2O_5	170 °C 9 h	127.5 mAh g^{-1} at 0.2 C; 120.5 mAh g^{-1} at 5 C (83.2%, 1000 cycles)	NVPF@GN‖soft carbon 108 mAh g^{-1} at 0.5 C; 59.6 mAh g^{-1} at 5 C (74.5%, 500 cycles)
NVPF/C@3DG[44]	Hydrothermal-annealing method	V_2O_5	170 °C 9 h for hydrothermal and 700 °C 4 h for calcination	123.6 mAh g^{-1} at 0.2 C; 91.2 mAh g^{-1} at 5 C (82.9%, 1000 cycles)	
Zr-doped NVPF/C[45]	carbothermal reduction method	NH_4VO_3	500 rpm 6 h for ball	121.8 mAh g^{-1} at 0.2 C; 112.3 mAh g^{-1} at 5 C (83.6%, 1000 cycles)	

			milling and 700 ºC 15 min for calcination		
NVPF@rGO[46]	polyol-assisted hydrothermal high temperature calcination	NH_4VO_3	180 ºC 20 h for hydrothermal and 500 ºC 4 h for calcination	119 mAh g^{-1} at 0.1 C; 76 mAh g^{-1} at 5 C (78.2%, 1500 cycles)	
N-doped NVPF/C[47]	sol–gel method	NH_4VO_3	80 ºC for sol-gel process, 650 ºC 10 h for calcination	126 mAh g^{-1} at 0.5 C; 87 mAh g^{-1} at 40 C (60.4%, 1500 cycles)	
NVPF@C[48]	Solution-solid method	V_2O_5	100 ºC 12 h for hydrothermal and 700 ºC 4 h for calcination	121.5 mAh g^{-1} at 0.1 C; 99.2 mAh g^{-1} at 10 C (90.1%, 1000 cycles)	
N-doped NVPF[49]	Electrospinning method	NH_4VO_3	700 ºC 5 h for calcination	109.5 mAh g^{-1} at 0.1 C; 84.6 mAh g^{-1} at 20 C (87.8%, 1000 cycles)	
NVPF@rGO[50]	sol–gel method	NH_4VO_3	80 ºC for sol-gel	113 mAh g^{-1} at 0.5 C; 91 mAh g^{-1} at 30 C (10000 cycles)	NVPF@rGO ||NVP 90.7 mAh g^{-1} at 0.5 C; 76.2 mAh g^{-1} at

			process, 700 ºC 5 h for calcination		2 C (83%, 300 cycles)
N-doped NVPF@C[51]	membrane casting method	NH_4VO_3	800 ºC 12 for annealing the membrane, 750 ºC 8 h for calcination	116.1 mAh g^{-1} at 0.2 C; 90.1 mAh g^{-1} at 10 C (80.5%,5000 cycles)	
NVOPF/KB (this work)	mechanochemical synthesis	$NaVO_3$	RT, 30 min	142.2 mAh g^{-1} at 0.1 C; 112.7 mAh g^{-1} at 20 C (98%, 10000 cycles)	NVOPF/KB‖hard carbon 88 Wh kg^{-1}

Supplementary Table 9. Atomic coordinates, cation, and anion occupancies of as-synthesized $Na_3(VOPO_4)_2F$/KB composite

Element type	Fractional coordinates x	y	z	Mult.	Occu.	Uiso
Na1	-0.2644(2)	-0.2644(2)	0	8	0.821(6)	0.0176(1)
Na2	-0.3605(1)	-0.2445(3)	0	16	0.147(2)	0.0112(7)
V1	0	0	0.2001(3)	4	1	0.0132(2)
P1	0.5	0	0.25	4	1	0.0986(2)
F1	0	0	0	2	1	0.0042(9)
O1	0.3128(1)	0	0.1609(5)	16	1	0.0184(1)
O2	0	0	0.3535(2)	4	1	0.0021(3)

Supplementary Table 10. Multi-point BET results of bare NVOPF and NVOPF/8%KB

Samples	NVOPF	NVOPF/8%KB
Surface Area(m^2 g^{-1})	30.443	44.572
Pore Volume(mL g^{-1})	0.081	0.131
Pore Diameter(nm)	5.772	10.166

Supplementary Table 11. The fitting resistance values for the half cells (NVOPF and NVOPF/8%KB) with different charge-discharge states

**Charge-discharge status for half cells**	**Cathode**	**R_p (ohm)**	**R_{SEI} (ohm)**	**R_{ct} (ohm)**
**OCV**	Bare NVOPF	5.971	63.47	294.4
	NVOPF/8%KB	7.764	53.31	245.3
**Charged state (1 st)**	Bare NVOPF	6.017	30.32	147.3
	NVOPF/8%KB	6.308	29.48	137.3

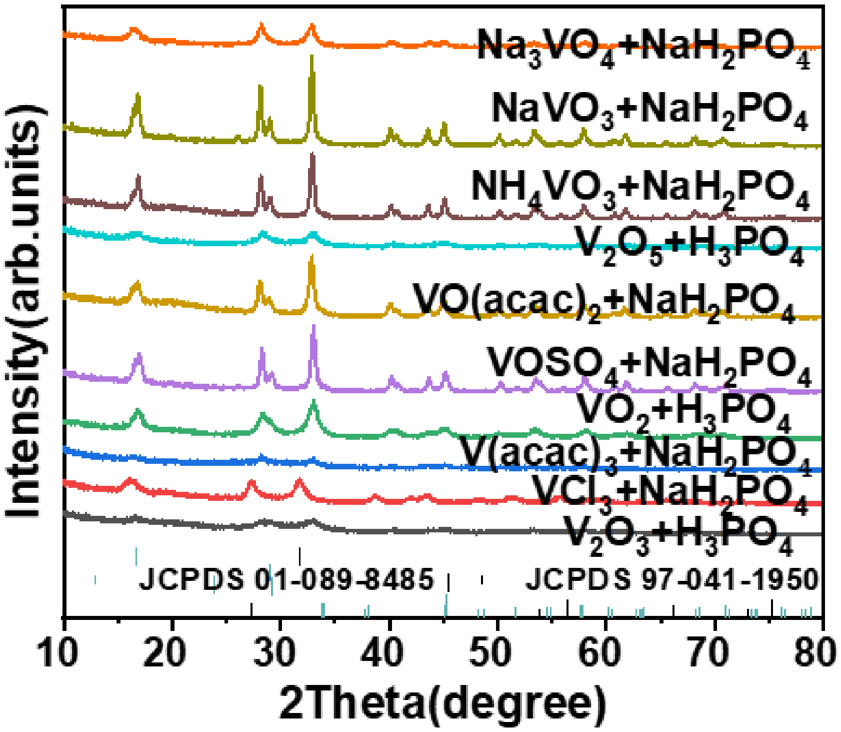

Supplementary Fig. 1 X-ray diffraction patterns of the successful preparation derived from ten different precursors

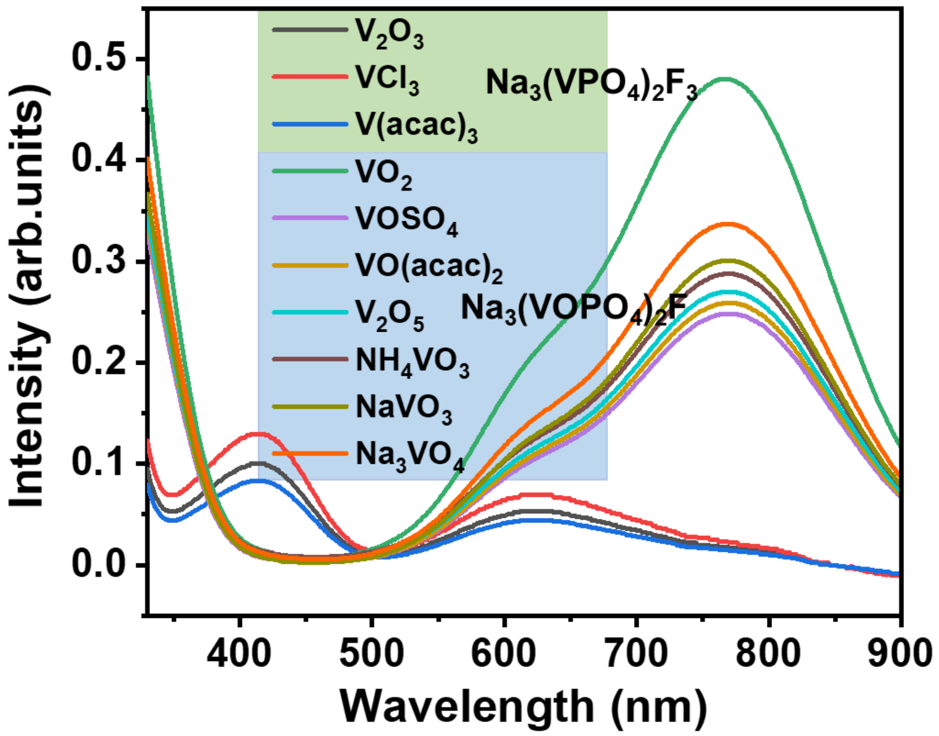

Supplementary Fig. 2 UV-vis spectra of the successful preparation derived from ten different precursors

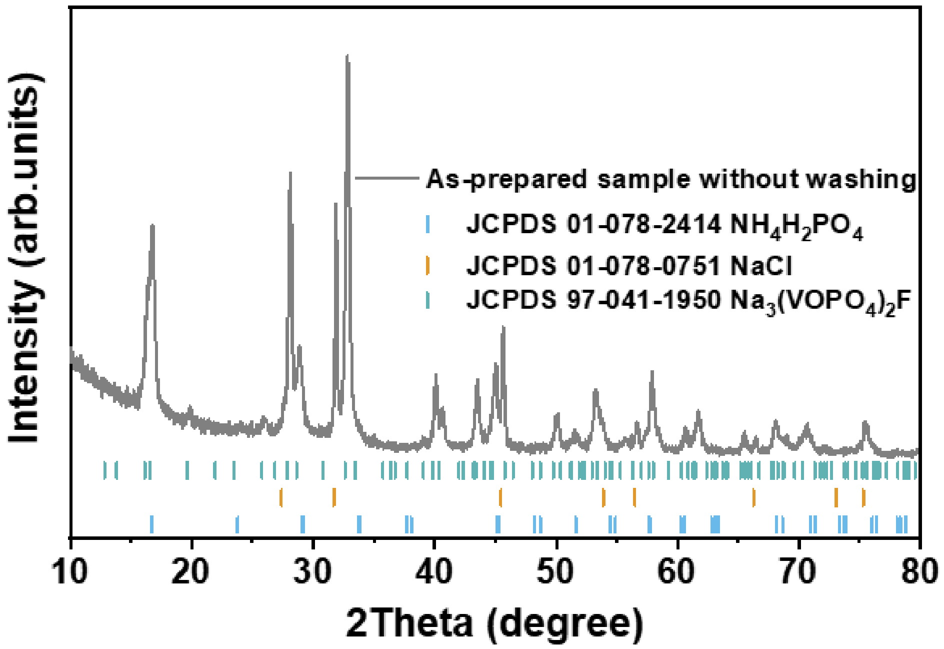

Supplementary Fig. 3 The XRD pattern of as-synthesized sample without washing obtained from $NaVO_3$ as precursor

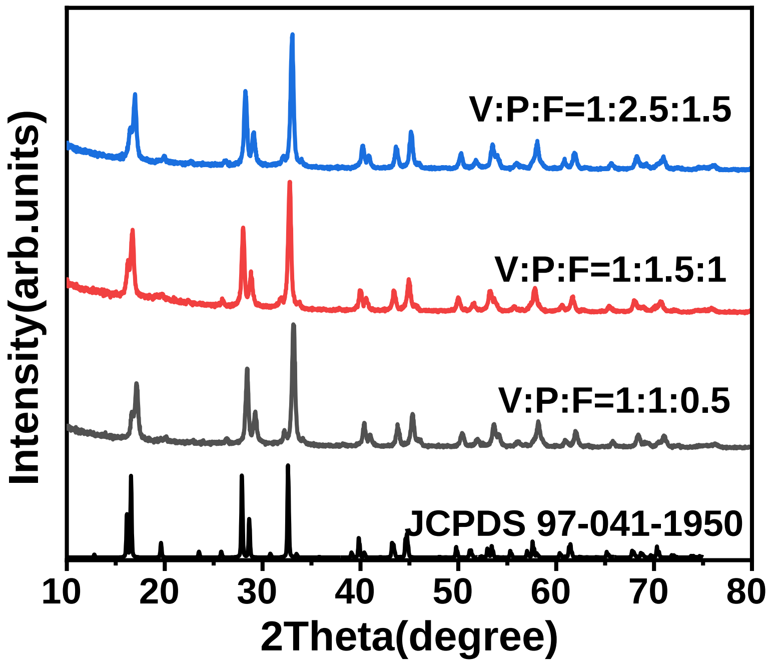

Supplementary Fig. 4 XRD patterns of samples obtained at different ratios of V, P and F from starting materials (V, P and F represents $NaVO_3$, $NaH_2PO_4 \cdot 2H_2O$ and NaF, respectively)

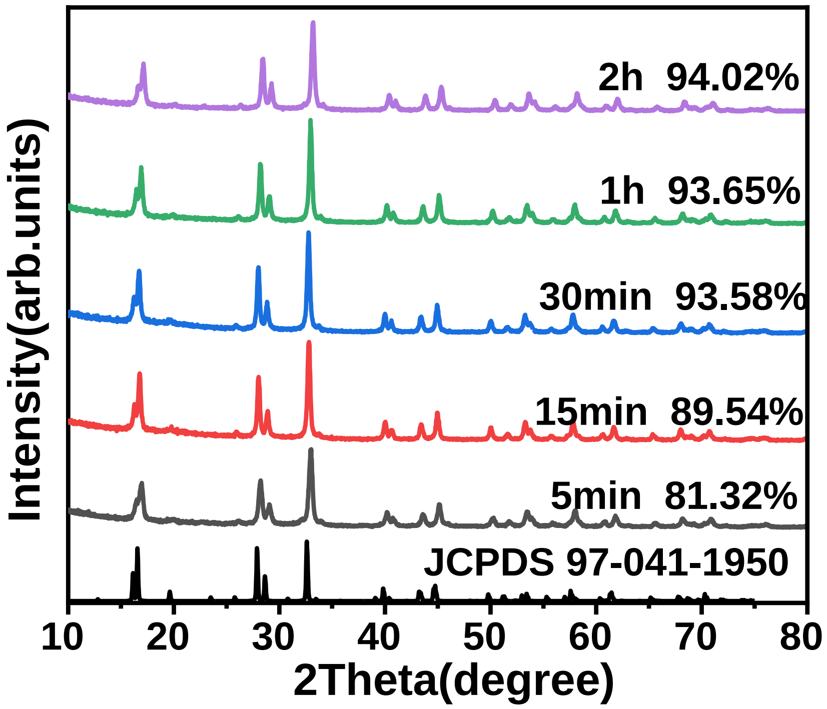

Supplementary Fig. 5 XRD patterns and yields of samples obtained from $NaVO_3$, $NaH_2PO_4 \cdot 2H_2O$ and NaF using different reaction time

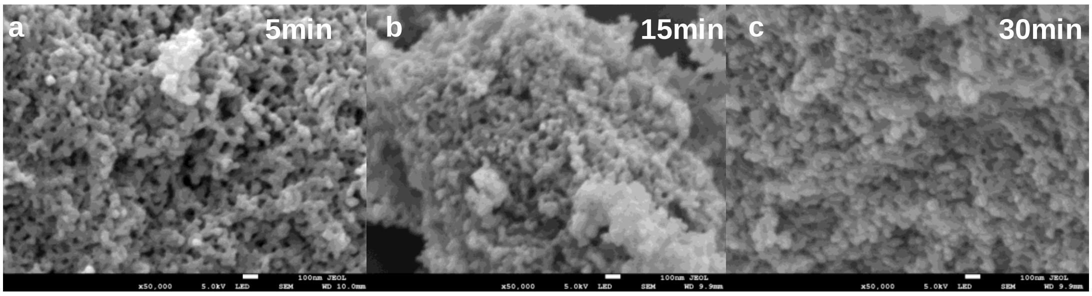

Supplementary Fig. 6 Typical SEM images of samples obtained at different reaction time

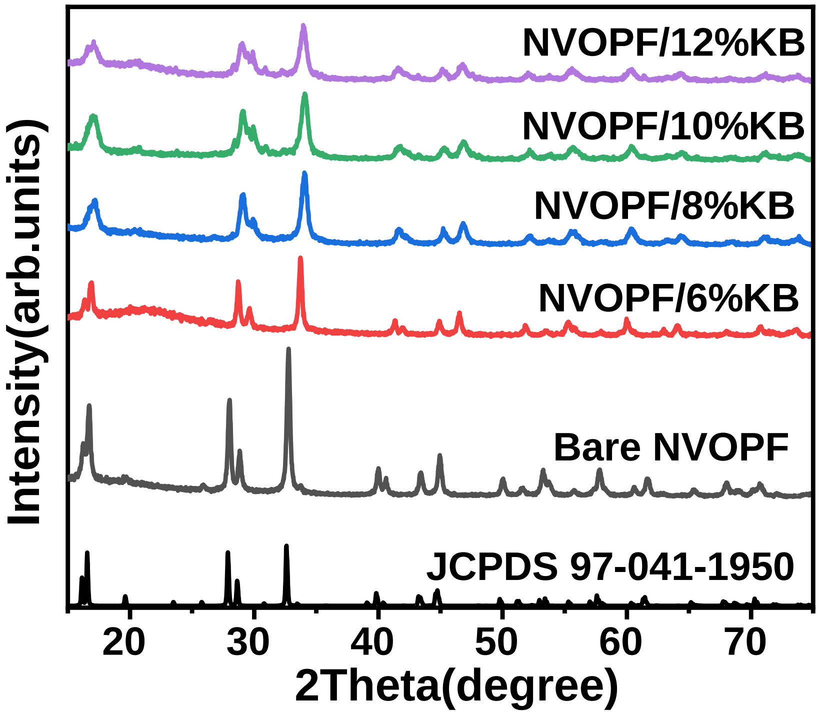

Supplementary Fig. 7 XRD patterns of as-synthesized NVOPF and in-situ constructed NVOPF/KB

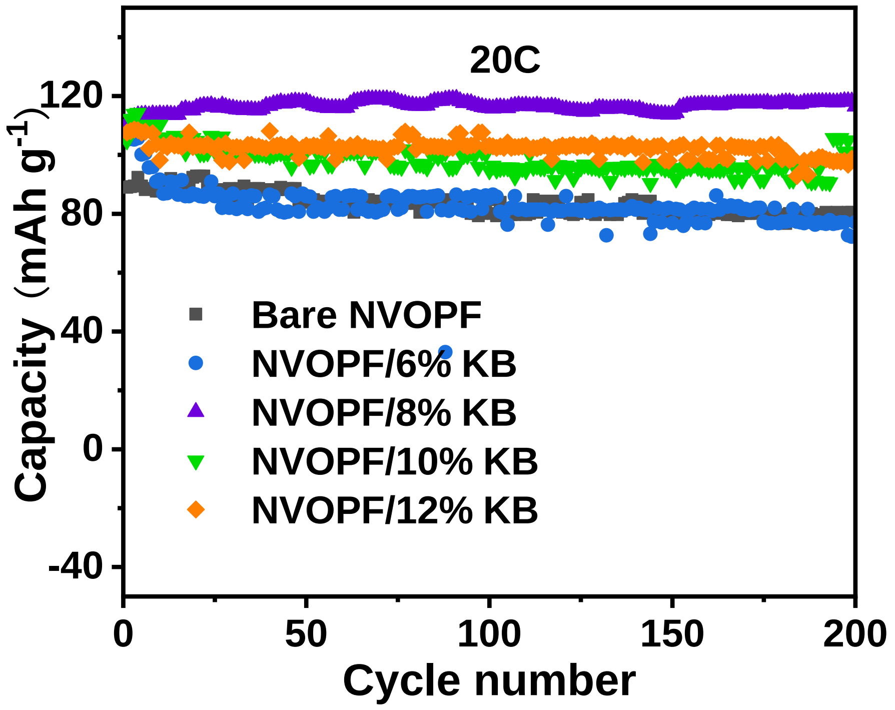

Supplementary Fig. 8 Cycling performances of NVOPF with increasing KB content

The excess capacity contribution

Firstly, we reconfirmed the discharge capacity with using five batches of NVOPF/8%KB samples with an active loading amount of 6.5 mg cm^{-2} (**Supplementary Fig. 9a**). Then, we observed the discharged KB with and without ball-milling to reveal the corresponding Na-storage properties. It can be found that the discharged capacity of KB with and without ball-milling are 3.5 and 0.7 mAh g^{-1}, respectively, which proves the Na-storage of ball-milled KB itself (**Supplementary Fig. 9b-c**). The X-ray photoelectron spectroscopy was employed to detect the chemical state of discharged KB electrodes, as shown in **Supplementary Fig. 9d**. No Na signal was observed in the discharged KB without ball-milling, but an obvious Na *1s* peak located at ~1069.2 eV can be found in the discharged KB with ball-milling, which confirms the binding effect between Na and KB. From this result, we can locate the Na-C binding energy to fit the following Na *1s* spectra. After this, we collected the Na *1s* XPS data of NVOPF and NVOPF/8%KB electrodes with varied charge/discharge states. As shown in **Supplementary Fig. 10a**, there was no obvious change in the Na peak for NVOPF during the first charge-discharge process, declaring only Na in the crystal structure can be migrated in the electrochemical reaction. Thus, the only Na peak can be indexed to the Na in NVOPF crystal structure, and it can not be distinguished between Na1 and Na2 shown in **Fig. 2b.** Whereas for NVOPF/8%KB, the extra Na-C peak can be detected in the pristine electrode, implying that Na^+ could transfer from NVOPF to carbon, forming Na–C bond in the ball-milled process. After charged to 4.2 V, the Na-C peak disappeared as the extraction of Na^+ from the NVOPF/8%KB species. When the NVOPF/8%KB electrode was discharged to 2.5 V, an extra Na peak emerged at 1071.7 eV, which denotes the formation of new Na-binding at this state, as seen from **Supplementary Fig. 10b**. The new Na-binding is not derived from the bulk of NVOPF or KB, but could be derived from the interfacial between NVOPF and KB. Owing to the nano-ionics properties, the interfacial Na is rather different from the homogeneous storage i.e., in NVOPF where Na^+ and e^- are inserted into a host lattice. In 2003, Maier et al. proposed the nanocrystallinity effects in Li-ion materials and the excess interfacial storage mechanism named charge separation at phase boundaries.[52,53] Inspired by the

extra Li-storage, the current interfacial Na-storage can be illustrated in **Supplementary Fig. 11**. In the interface between $Na_3(VOPO_4)_2F$ and KB, Na^+ can be accommodated at the boundary of NVOPF side while the electrons are restricted to the KB side. For the combination of $Na_3(VOPO_4)_2F$ and KB, the stored Na^+ and e^- act as a bridge during the charge-discharge process. With nanoscale material properties, it forms a bridge between batteries and capacitors in the mesoscopic case, an interfacial storage can be expected.

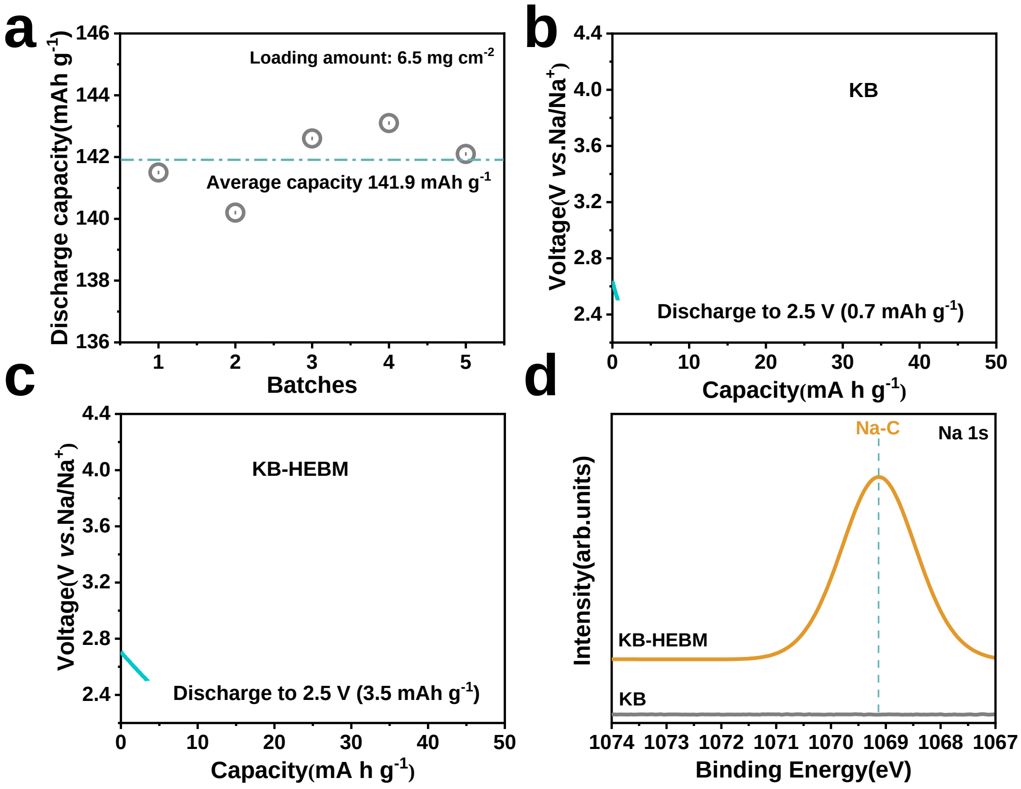

Supplementary Fig. 9 (a) The first-discharge specific capacity of 5 batches of NVOPF/8%KB cathode samples. The discharge curves of KB from OCV (b) without and (c) with ball-milling process at the current rate of 0.1 C. (d) XPS Na 1s spectra of discharged KB electrodes with and without ball-milling.

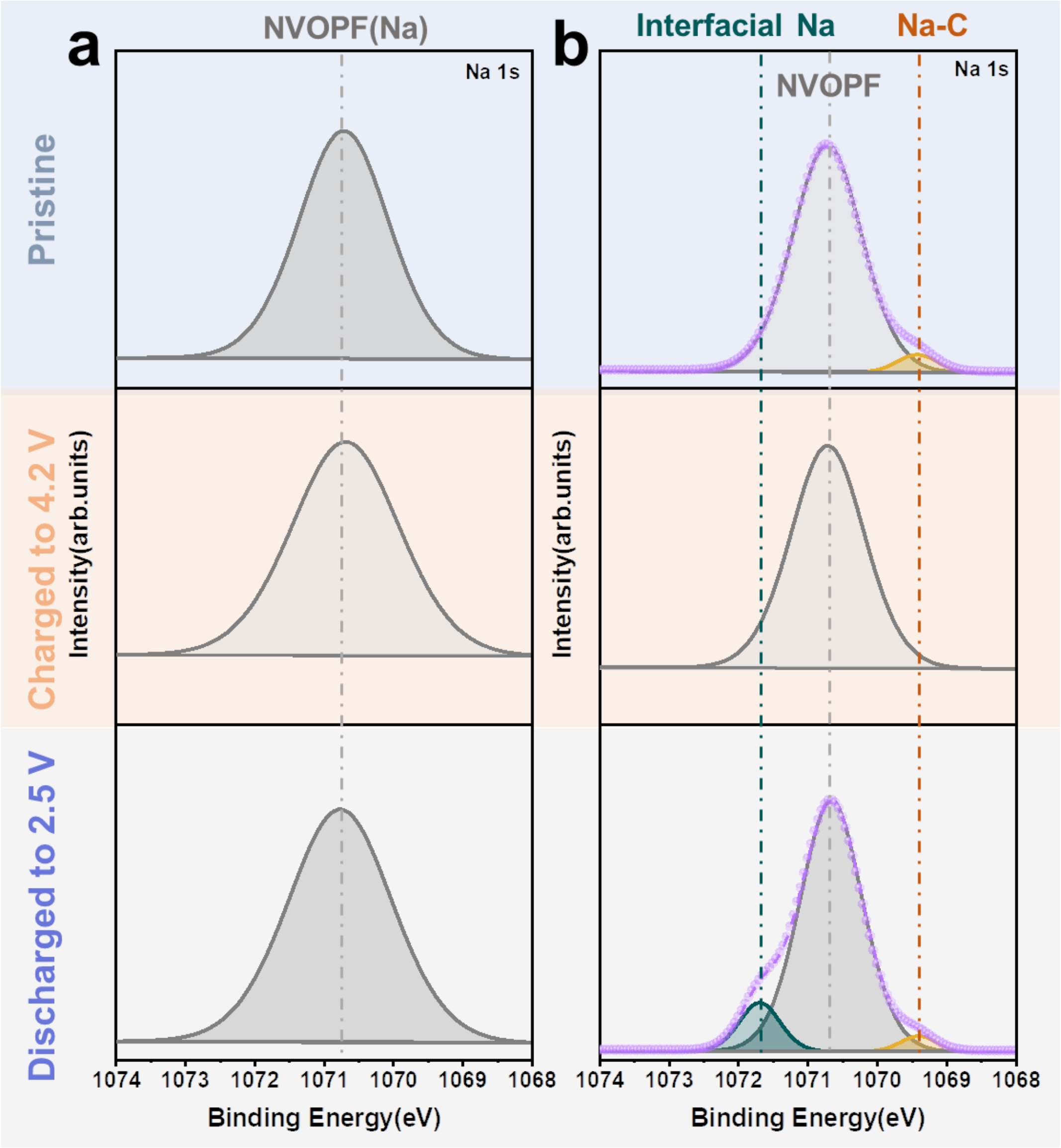

Supplementary Fig. 10 XPS Na *1s* spectra of (a) NVOPF and (b) NVOPF/8%KB with varied charge-discharge states

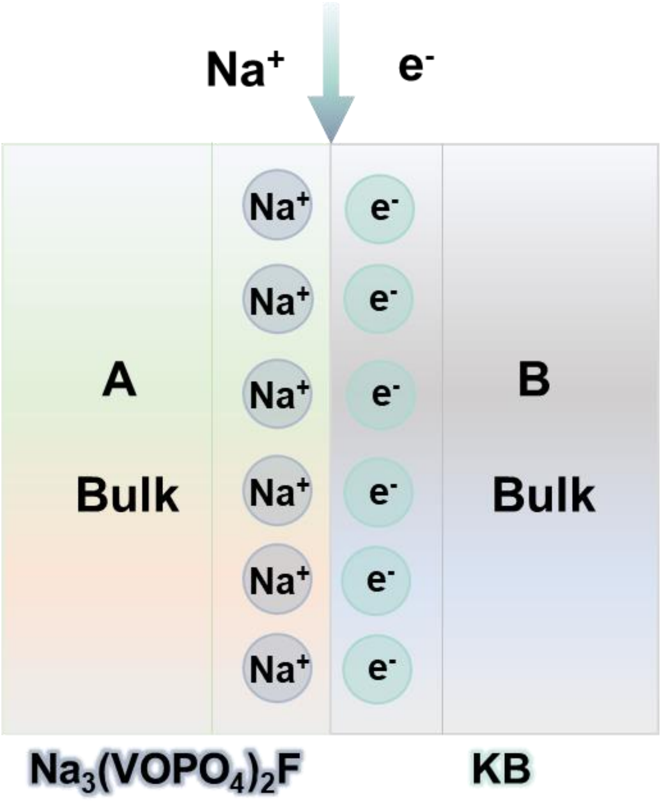

Supplementary Fig. 11 The schematic diagram of NVOPF/8%KB interfacial Na-storage mechanism based on charge separation at phase boundaries

Post-mortem analysis of NVOPF/8%KB cathode

Post-mortem analysis of NVOPF/8%KB cathode laminate after 11386 cycles (decay to the point with no capacity) was employed. It could be seen that the separator was permeated with brown compounds due to the violent redox reactions in the electrolyte. In this process, the crystal structure of NVOPF/KB electrode was not vandalized (**Supplementary Fig. 12c**), which further confirms the structure stability of the *in-situ* constructed composites.

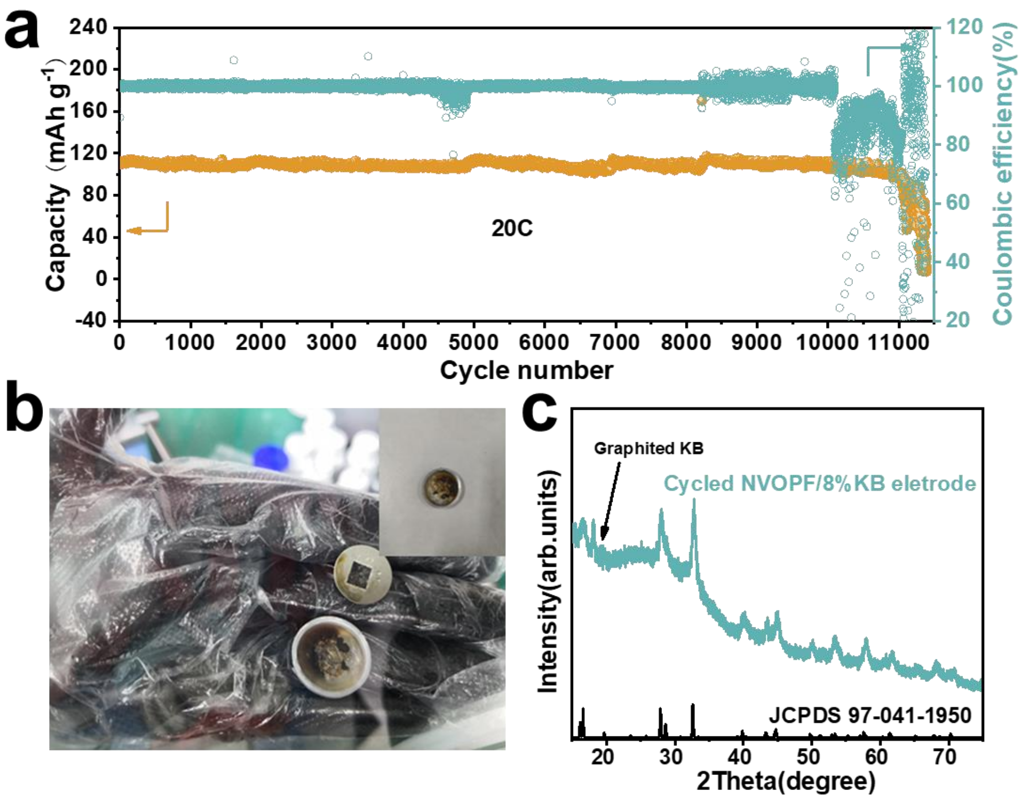

Supplementary Fig. 12 (a) The cycling performance of NVOPF/8% KB electrode over 11386 times at 20 C. (b) The digital image of postmortem electrode cycled after 11386 cycles. (c) The XRD pattern of NVOPF/8%KB cathode cycled for 11386 cycles.

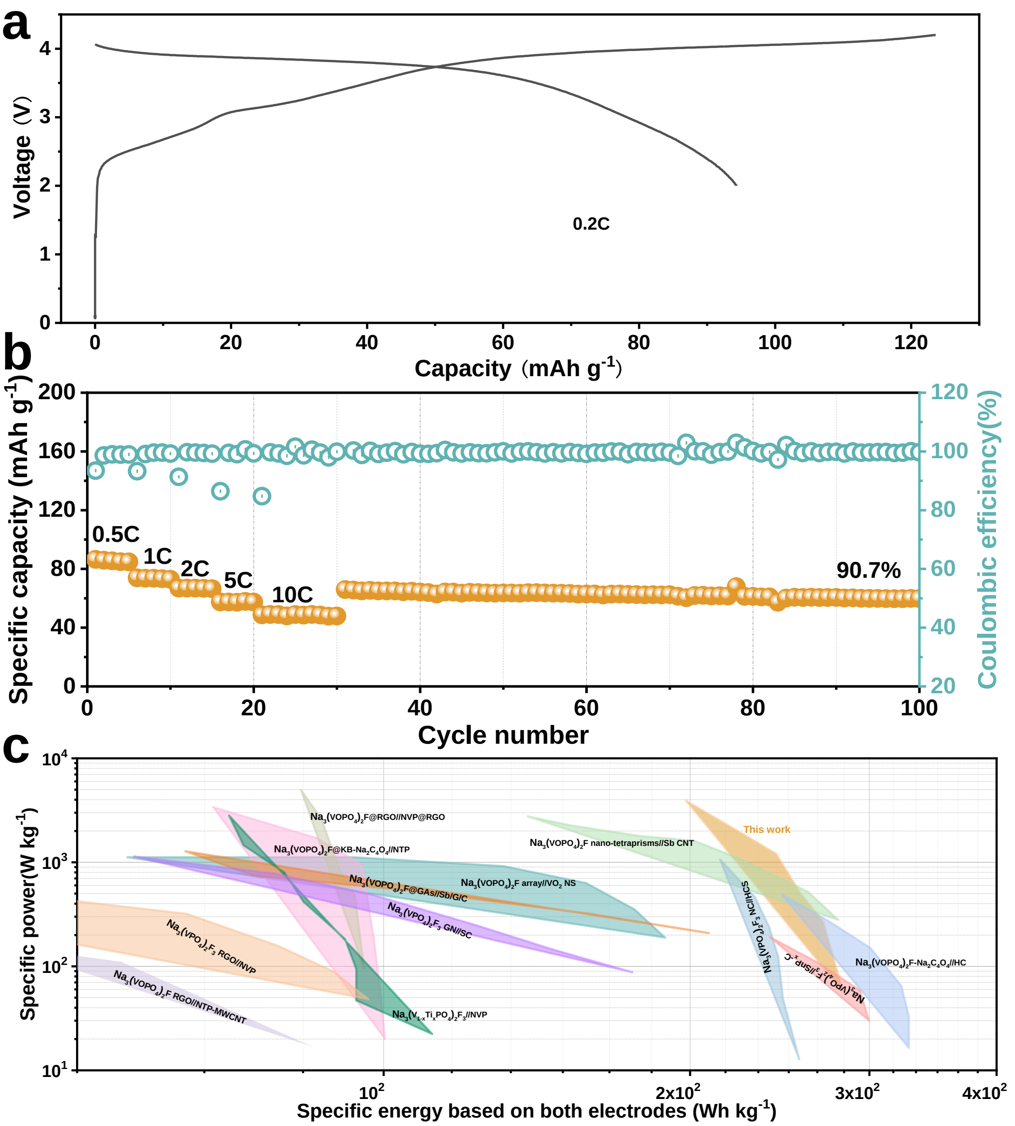

Supplementary Fig. 13 (a) The charge-discharge curves of the coin full cell for the first cycle at 0.2 C. (b) Rate capability of full cells at various rates from 0.5 C to 10 C. (c) The Ragone plots of NVPF analogous compounds.

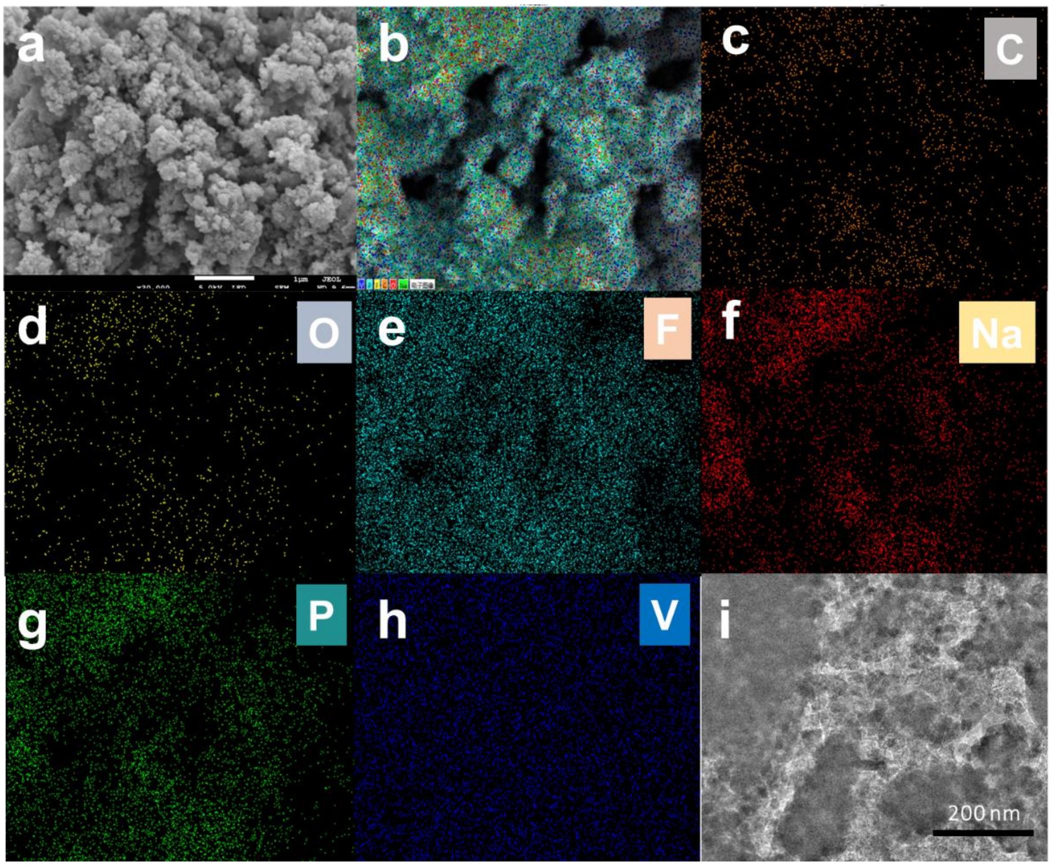

Supplementary Fig. 14 The SEM image (a), overall element distribution (b), corresponding elemental mapping images for C (c), O (d), F (e), Na (f), P (g), V (h) and TEM image of in-situ constructed NVOPF/8%KB cathode (i)

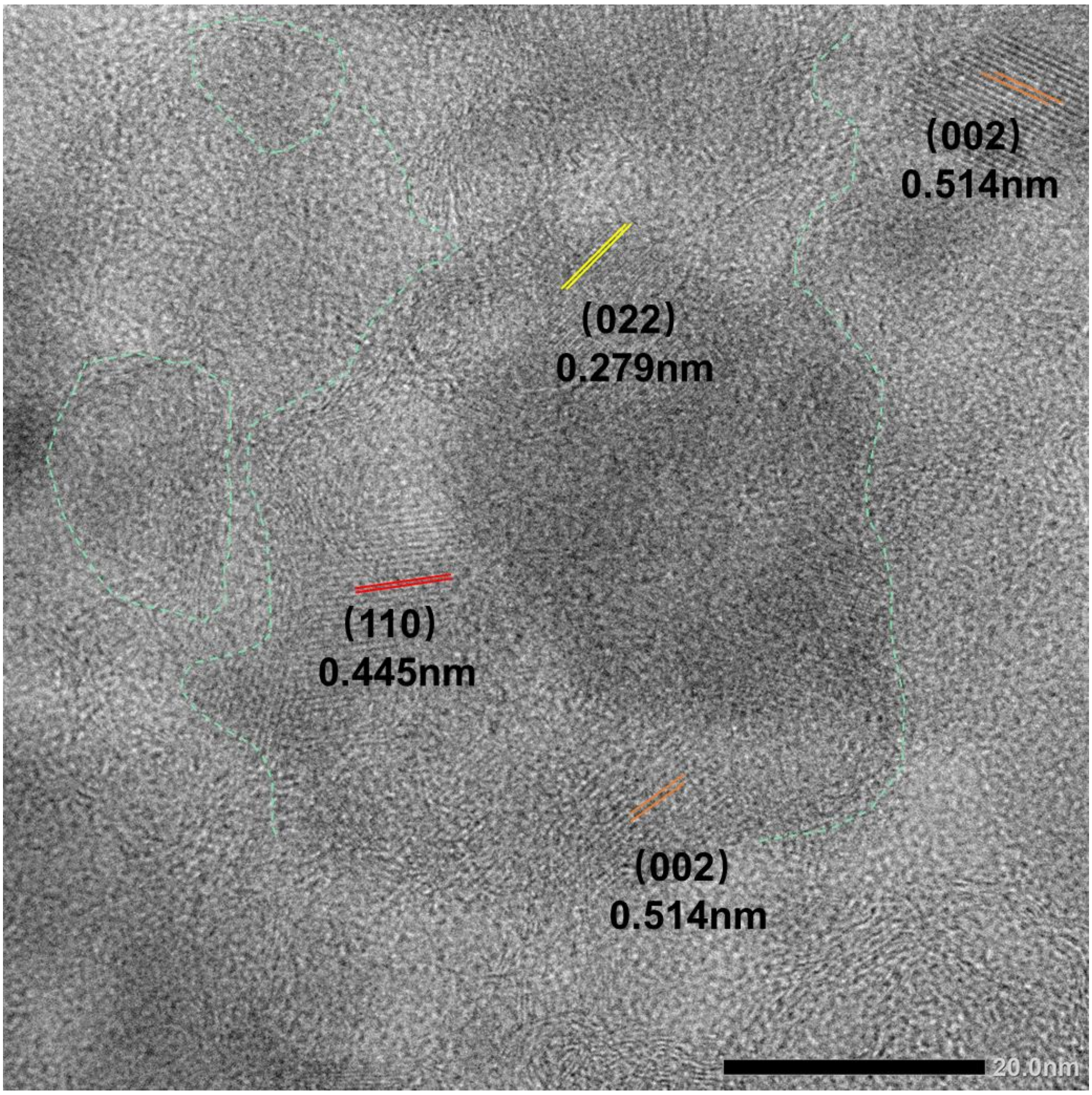

Supplementary Fig. 15 HRTEM image and four exposed crystal planes of *in-situ* constructed NVOPF/8%KB composite

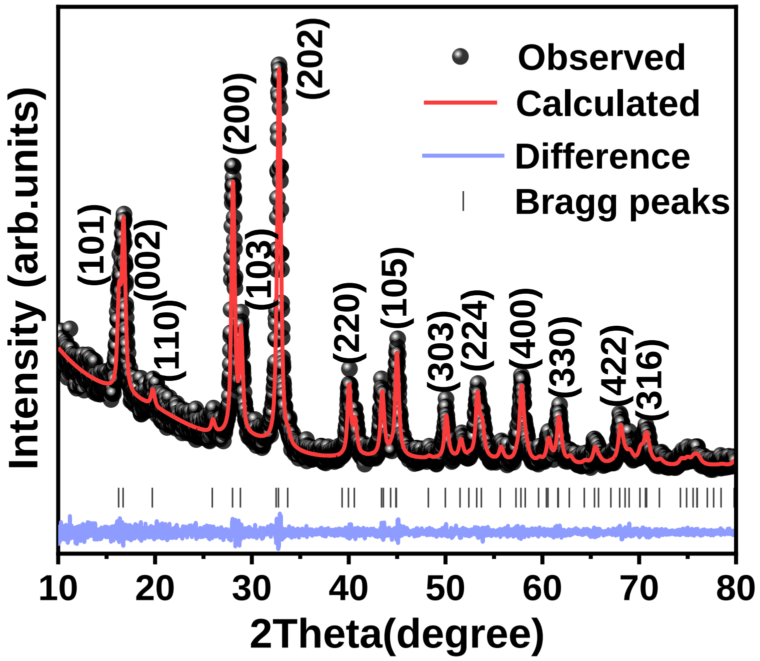

Supplementary Fig. 16 Rietveld-refined profiles of as-synthesized NVOPF/KB (The unit cell parameters are a = b = 6.3896 Å and c=10.6601 Å with good reliability factor values (Rwp =6.37%, Rp =8.37%, x^2=2.15), which presents a larger lattice volume of 435.22 $Å^3$ than bare NVOPF (434.98 $Å^3$).

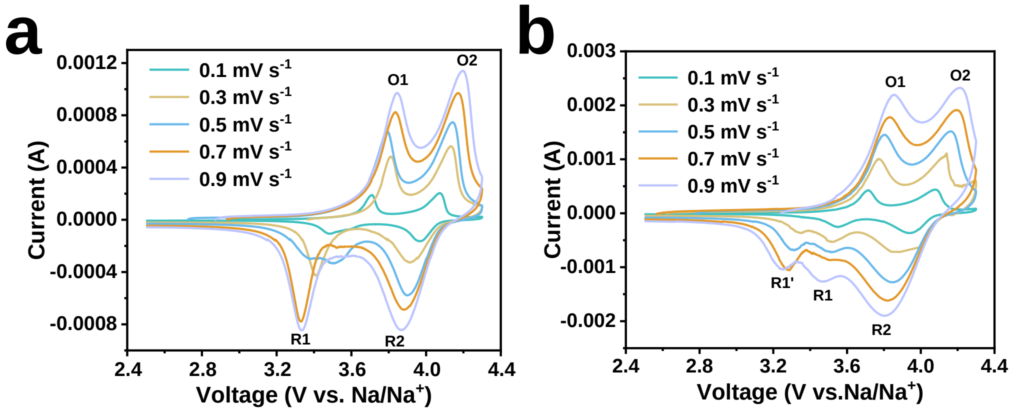

Supplementary Fig. 17 CV profiles of bare NVOPF (a) and NVOPF/8%KB (b) electrodes at various scan rates from 0.1 to 0.9 mV s^{-1}.

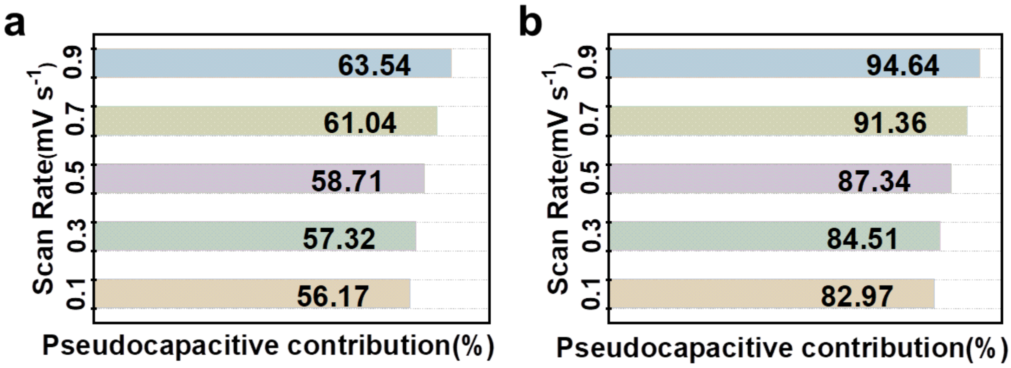

Supplementary Fig. 18 The corresponding pseudocapacitive contributions of bare NVOPF (a) and NVOPF/8%KB (b) at different scan rates

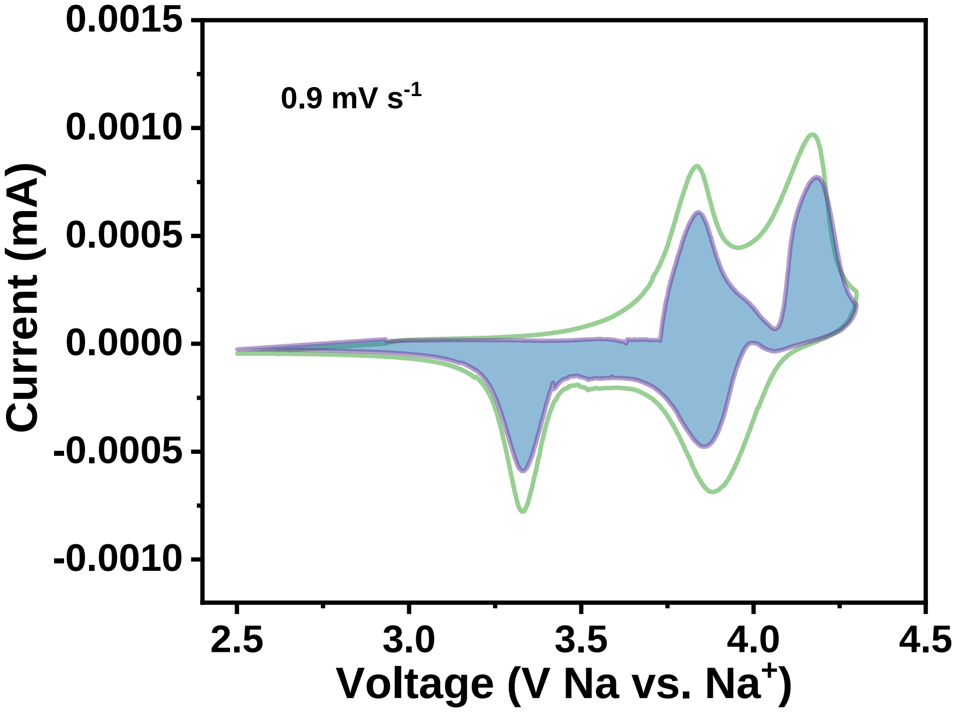

Supplementary Fig. 19 CV curve of bare NVOPF with the pseudocapacitive fraction shown by the blue region at a scan rate of 0.9 mV s^{-1}

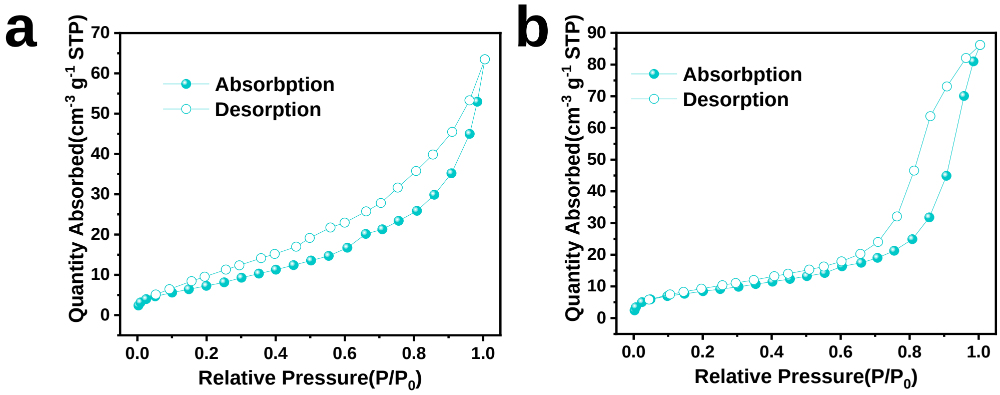

Supplementary Fig. 20 The nitrogen adsorption−desorption isotherms of the bare NVOPF (a) and NVOPF/8%KB (b)

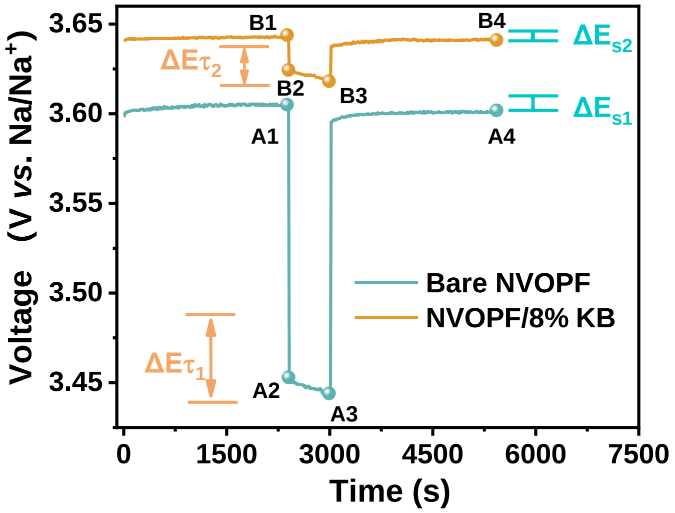

Supplementary Fig. 21 Comparison of a single GITT titration between the bare NVOPF and NVOPF/8%KB electrodes

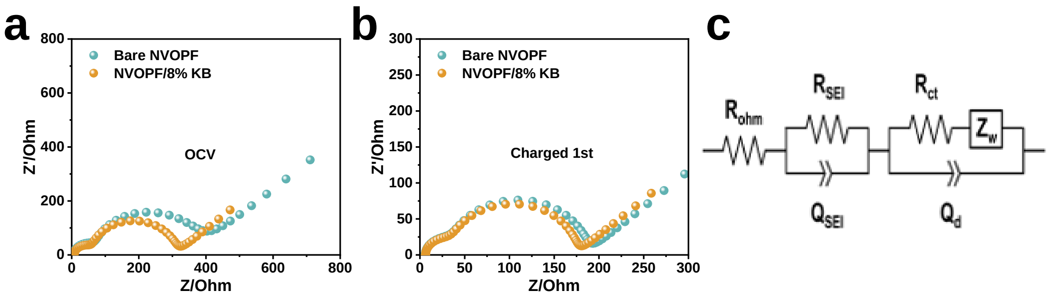

Supplementary Fig. 22 The Nyquist plots of half cells prior to cycling (a) and charged state after one cycle (b). (c) The equivalent circuit for fitted Nyquist plots.

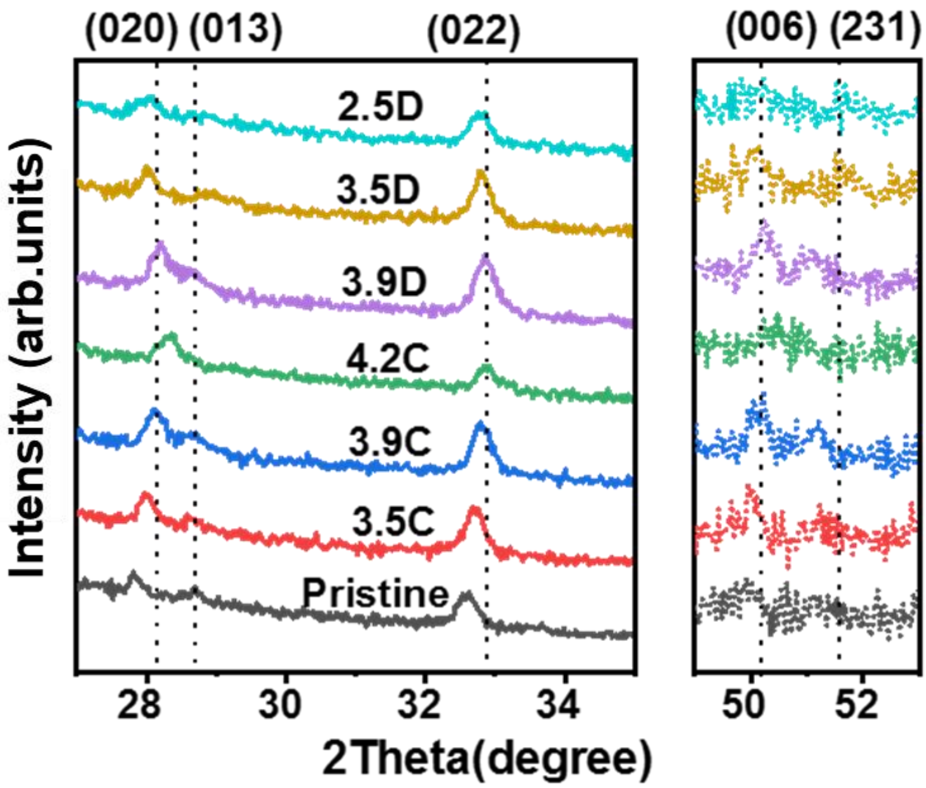

Supplementary Fig. 23 *Ex-sit*u XRD patterns of bare NVOPF cathode

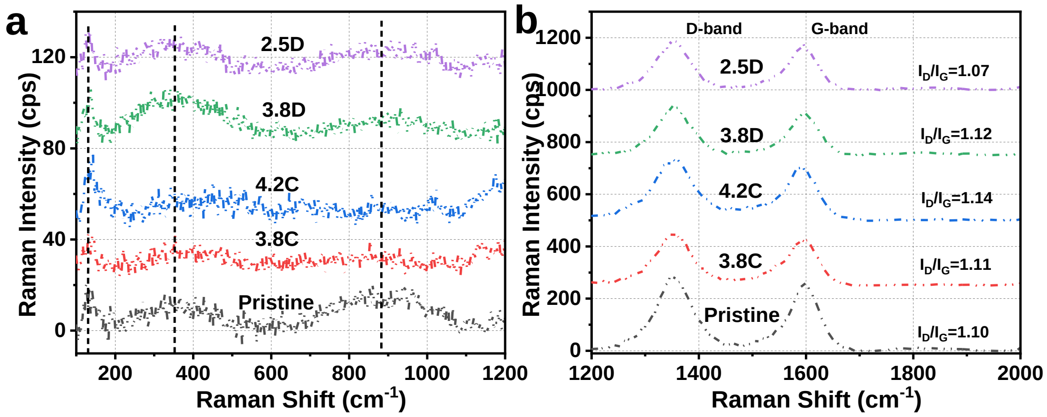

Supplementary Fig. 24 *Ex-situ* Raman spectra of NVOPF/8%KB electrodes cycled at various states: 200-1200 cm^{-1} (a) and 1200-2000 cm^{-1} (b)

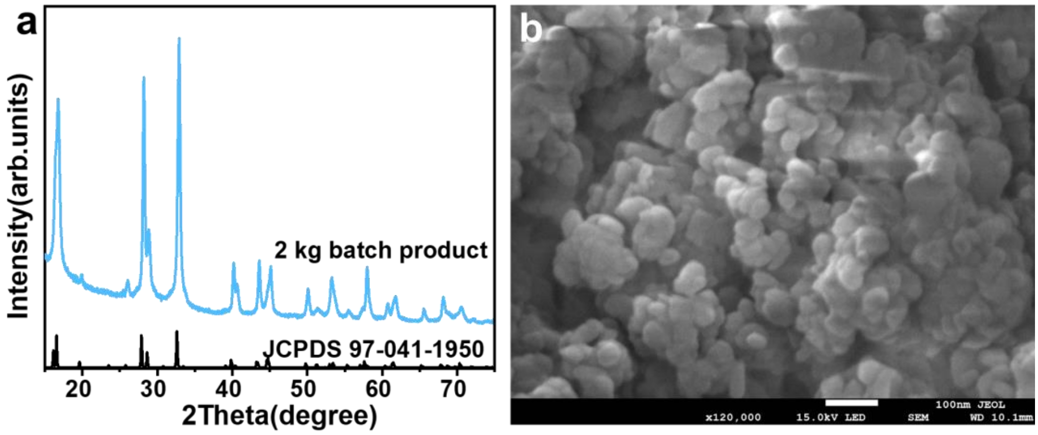

Supplementary Fig. 25 The powder XRD pattern and SEM image of the scale-up products of NVOPF/KB composites

Supplementary References

1 Sauvage, F., Quarez, E., Tarascon, J. M. & Baudrin, E. Crystal structure and electrochemical properties vs. Na^+ of the sodium fluorophosphate $Na_{1.5}VOPO_4F_{0.5}$. *Solid State Sci.* **8**, 1215-1221 (2006).

2 Serras, P., Palomares, V., Kubiak, P., Lezama, L. & Rojo, T. Enhanced electrochemical performance of vanadyl (IV) $Na_3(VO)_2(PO_4)_2F$ by *ex-situ* carbon coating. *Electrochem. Commun.* **34**, 344-347 (2013).

3 Serras, P., Palomares, V., Rojo, T., Brand, H. E. A. & Sharma, N. Structural evolution of high energy density V^{3+}/V^{4+} mixed valent $Na_3V_2O_{2x}(PO_4)_2F_{3-2x}$ (x=0.8) sodium vanadium fluorophosphate using in situ synchrotron X-ray powder diffraction. *J. Mater. Chem. A* **2**, 7766-7779 (2014).

4 Zhao, J., Mu, L., Qi, Y., Hu, Y.-S., Liu, H. & Dai, S. A phase-transfer assisted solvo-thermal strategy for low-temperature synthesis of $Na_3(VO_{1-x}PO_4)_2F_{1+2x}$ cathodes for sodium-ion batteries. *Chem. Commun.* **51**, 7160-7163 (2015).

5 Peng, M. *et al.* Ruthenium-oxide-coated sodium vanadium fluorophosphate nanowires as high-power cathode materials for sodium-ion batteries. *Angew. Chem. Int. Edit.* **54**, 6452-6456 (2015).

6 Qi, Y., Mu, L., Zhao, J., Hu, Y.-S., Liu, H. & Dai, S. Superior Na-storage performance of low-temperature-synthesized $Na_3(VO_{1-x}PO_4)_2F_{1+2x}$ ($0 \leq x \leq 1$) nanoparticles for Na-ion batteries. *Angew. Chem. Int. Edit.* **54**, 9911-9916 (2015).

7 Gang, D. et al. Graphene quantum dots-shielded $Na_3(VO)_2(PO_4)_2F$@C nanocuboids as robust cathode for Na-ion battery. *Energy Storage Mater.* **5**, 198-204 (2016).

8 Guo, J.-Z. et al. High-energy/power and low-temperature cathode for sodium-ion batteries: In situ XRD study and superior full-cell performance. *Adv. Mater.* **29**, 1701968 (2017).

9 Kumar, P. R., Young Hwa, J., Syed Abdul, A. & Do Kyung, K. A high rate and stable electrode consisting of a $Na_3V_2O_{2X}(PO_4)_2F_{3-2X}$–rGO composite with a cellulose binder for sodium-ion batteries. *RSC Adv.* **7**, 21820-21826 (2017).

10 Peng, M. et al. Hierarchical Ru-doped sodium vanadium fluorophosphates hollow microspheres as a cathode of enhanced superior rate capability and ultralong stability for sodium-ion batteries. *Nano Energy* **31**, 64-73 (2017).

11 Yin, Y.-M. et al. Robust three-dimensional graphene skeleton encapsulated $Na_3V_2O_2(PO_4)_2F$ nanoparticles as a high-rate and long-life cathode of sodium-ion batteries. *Nano Energy* **41**, 452-459 (2017).

12 Chao, D. et al. Sodium vanadium fluorophosphates (NVOPF) array cathode designed for high-rate full sodium ion storage device. *Adv. Energy Mater.* **8**, 1800058 (2018).

13 Liu, K., Lei, P., Xin, W., Zheng, W. & Xiang, X. Cost-effective synthesis and superior electrochemical performance of sodium vanadium fluorophosphate nanoparticles encapsulated in conductive graphene network as high-voltage cathode for sodium-ion batteries. *J. Colloid Interf. Sci.* **532**, 426-432 (2018).

14 Shanmukaraj, D. et al. Highly efficient, cost effective, and safe sodiation agent for high-performance sodium-ion batteries. *Chemsuschem* **11**, 3286-3291 (2018).

15 Verónica, P. et al. Waste biomass as in situ carbon source for sodium vanadium fluorophosphate/C cathodes for Na-ion batteries. *Acs Sustainable Chem. Eng.* **12**, 16386-16398 (2018).

16 Qi, Y. et al. Scalable room-temperature synthesis of multi-shelled $Na_3(VOPO_4)_2F$ microsphere cathodes. *Joule* **2**, 2348-2363 (2018).

17 Qi, Y., Zhao, J., Yang, C., Liu, H. & Hu, Y.-S. Comprehensive studies on the hydrothermal strategy for the synthesis of $Na_3(VO_{1-x}PO_4)_2F_{1+2x}$ ($0\leq x\leq 1$) and their Na-Storage performance. *Small Methods* **3**, 1800111 (2019).

18 Gu, Z.-Y. et al. Precisely controlled preparation of an advanced $Na_3V_2(PO_4)_2O_2F$ cathode material for sodium ion batteries: the optimization of electrochemical properties and electrode kinetics. *Inorg. Chem. Front.* **6**, 988-995 (2019).

19 Hou, Y. et al. Rapid microwave-assisted refluxing synthesis of hierarchical mulberry-shaped $Na_3V_2(PO_4)_2O_2F$@C as high performance cathode for sodium

& lithium-ion batteries. *Sci. China Mater.* **62**, 474-486 (2019).

20 Nguyen, L. H. B. et al. Monitoring the crystal structure and the electrochemical properties of $Na_3(VO)_2(PO_4)_2F$ through Fe^{3+} Substitution. *Acs Appl. Mater. Inter.* **11**, 38808-38818 (2019).

21 Du, P., Li, T., Jiang, X., Wang, D. & Zheng, X. Improving the electrochemical performance of $Na_3V_2O_2(PO_4)_2F$ cathode by using a defect-containing TiO_{2-x} coating for sodium ion batteries. *J. Alloy. Compd.* **814**, 152270 (2019).

22 Shen, X. et al. Controlled synthesis of $Na_3(VOPO_4)_2F$ cathodes with an ultralong cycling performance. *Acs Appl. Energy Mater.* **2**, 7474-7482 (2019).

23 Zhang, Z. et al. Toward high power-high energy sodium cathodes: A case study of bicontinuous ordered network of 3D porous $Na_3(VO)_2(PO_4)_2F$/rGO with pseudocapacitance effect. *Small* **15**, 1900356 (2019).

24 Mao, Z., Wang, R., He, B., Gong, Y.-S. & Wang, H. Large-area, uniform, aligned arrays of $Na_3(VO)_2(PO_4)_2F$ on carbon nanofiber for quasi-solid-state sodium-ion hybrid capacitors. *Small* **15**, 1902466 (2019).

25 Tong, Z. et al. One-Step synthesis of carbon-coated $Na_3(VOPO_4)_2F$ using biomass as a reducing agent and their electrochemical properties. *Waste Biomass Valori.* **11**, 2201-2209 (2018).

26 Palomares, V. et al. Iron-doped sodium–vanadium fluorophosphates: $Na_3V_{2-y}O_{2-y}Fe_y(PO_4)_2F_{1+y}$ ($y < 0.3$). *Inorg. Chem.* **59**, 854-862 (2020).

27 Wei, C. et al. Freestanding $Na_3V_2O_2(PO_4)_2F$/Graphene aerogels as high-performance cathodes of sodium-ion full batteries. *Acs Appl. Mater. Inter.* **37**, 41419-41428 (2020).

28 Qiang, L. et al. Carbon-coated $Na_3V_2(PO_4)_2F_3$ nanoparticles embedded in a mesoporous carbon matrix as a potential cathode material for sodium-ion batteries with superior rate capability and long-term cycle life. *J. Mater. Chem. A* **3**, 21478-21485 (2015).

29 Zhu, C. et al. A high power–high energy $Na_3V_2(PO_4)_2F_3$ sodium cathode: Investigation of transport parameters, rational design and realization. *Chem. Mater.* **29**, 5207-5215 (2017).

30 Li, L., Xu, Y., Sun, X., He, S. & Li, L. High capacity-favorable tap density cathode material based on three-dimensional carbonous framework supported $Na_3V_2(PO_4)_2F_3$ nanoparticles. *Chem. Eng. J.* **331**, 712-719 (2017).

31 Yi, H. et al. Scalable and economic synthesis of high-performance $Na_3V_2(PO_4)_2F_3$ by a solvothermal–ball-milling method. *Acs Energy Lett.* **4**, 1565-1571 (2019).

32 Nicolas, E. et al. Sodium vanadium (III) fluorophosphate/carbon nanotubes composite (NVPF/CNT) prepared by spray-drying: good electrochemical performance thanks to well-dispersed CNT network within NVPF particles. *Electrochim. Acta* **228**, 319-324 (2017).

33 Liu, W., Yi, H., Zheng, Q., Li, X. & Zheng, H. Y-doped $Na_3V_2(PO_4)_2F_3$ compounds for sodium ion battery cathode: electrochemical performance and analysis of kinetic properties. *J. Mater. Chem. A*, **5**, 10928-10935 (2017).

34 Ma, D. et al. Enhanced electrochemical performance of carbon and aluminum oxide co-coated $Na_3V_2(PO_4)_2F_3$ cathode material for sodium ion batteries. *Electrochim. Acta*, **283**, 1441-1449 (2018).

35 Yi, H. et al. VSC-doping and VSU-doping of $Na_3V_{2-x}Ti_x(PO_4)_2F_3$ compounds for sodium ion battery cathodes: Analysis of electrochemical performance and kinetic properties. *Nano Energy* **47**, 340-352 (2018).

36 Zhang, L. et al. Polydopamine-derived nitrogen-doped carbon-covered $Na_3V_2(PO_4)_2F_3$ cathode material for high-performance Na-Ion batteries. *Acs Appl. Mater. Inter.* **10**, 36851-36859 (2018).

37 Li, Y. et al. In-situ constructing $Na_3V_2(PO_4)_2F_3$/carbon nanocubes for fast ion diffusion with high-performance Na^+-storage. *Chem. Eng. J.* **387**, 123952 (2019).

38 Li, L., Liu, X., Tang, L., Liu, H. &Wang, Y-G. Improved electrochemical performance of high voltage cathode $Na_3V_2(PO_4)_2F_3$ for Na-ion batteries through potassium doping. *J. Alloy. Compd.* **790**, 203-211 (2019).

39 Wang, M. et al. Synthesis and electrochemical performances of $Na_3V_2(PO_4)_2F_3$/C composites as cathode materials for sodium ion batteries. *RSC*

Adv. **9**, 30628-30636 (2019).

40 Ayan, M., Tali, S., Rosy, S., Sivan, O. & Malachi, N. Effect of crystal structure and morphology on $Na_3V_2(PO_4)_2F_3$ performances for Na-Ion batteries. *Batteries & Supercaps* **3**, 510 (2020).

41 Hayong, S. & KwangSup, E. Overcoming the unfavorable kinetics of $Na_3V_2(PO_4)_2F_3$//SnP_x full-cell sodium-ion batteries for high specific energy and energy efficiency. *Adv. Funct. Mater.* **30**, 2003086 (2020).

42 Xun, J., Zhang, Y. & Xu, H. One step synthesis of vesicular $Na_3V_2(PO_4)_2F_3$ and network of $Na_3V_2(PO_4)_2F_3$@Graphene nanosheets with improved electrochemical performance as cathode material for sodium ion battery. *Inorg. Chem. Commun.* **115**, 107884 (2020).

43 Zhu, L. et al. Engineering the crystal orientation of $Na_3V_2(PO_4)_2F_3$@rGO microcuboids for advanced sodium-ion batteries. *Mater. Chem. Front.* **4**, 2932-2942 (2020).

44 Hu, L. et al. Dually decorated $Na_3V_2(PO_4)_2F_3$ by carbon and three-dimensional graphene as cathode material for sodium-ion batteries with high energy and power densities. *ChemElectroChem*, **7**, 3975 (2020).

45 Wang, M. et al. Improved sodium storage properties of Zr-doped $Na_3V_2(PO_4)_2F_3$/C as cathode material for sodium ion batteries. *Ceram. Int.* **46**, A28490-A28498 (2020).

46 Du, P. et al. Hierarchical hollow microspheres $Na_3V_2(PO_4)_2F_3C$@rGO as high-performance cathode materials for sodium ion batteries. *New J. Chem.* **44**, 12985-1299 (2020).

47 Wang, T. et al. N-Doped carbon nanotubes decorated $Na_3V_2(PO_4)_2F_3$ as a durable ultrahigh-rate cathode for sodium ion batteries. *Acs Appl. Energy Mater.* **3**, 3845-3853 (2020).

48 Zhan, W. et al. Ultra-long cycle life and high rate performance subglobose $Na_3V_2(PO_4)_2F_3$@C cathode and its regulation. *Int. J. Energy Res.* **44**, 6608-6622 (2020).

49 Li, Y. et al. Fiber-shape $Na_3V_2(PO_4)_2F_3$@N-doped carbon as a cathode material

with enhanced cycling stability for Na-ion batteries. *Acs Appl. Mater. Inter.* **12**, 25920-25929 (2020).

50 Yuvaraj, S. et al. Optimizing High voltage $Na_3V_2(PO_4)_2F_3$ cathode for achieving high rate sodium-ion batteries with long cycle life. *Chem. Eng. J.* **403**, 126291 (2020).

51 Lv, Z. et al. Electrode design for high-performance sodium-ion batteries: coupling nanorod-assembled $Na_3V_2(PO_4)3$@C microspheres with a 3D conductive charge transport network. *Acs Appl. Mater. Inter.* **12**, 13869-13877 (2020).

52 Jannik, J & Maier, J. Nanocrystallinity effects in lithium battery materials. (Aspects of nano-ionics. Part IV). *Phys. Chem. Chem. Phys.* **5**, 5215-5220 (2003).

53 Maier, J. Nanoionics: ion transport and electrochemical storage in confined systems. *Nat. Mater.* **4**, 805-815 (2005).
